# Biliary tract cancers: SEOM clinical guidelines

**DOI:** 10.1007/s12094-015-1436-2

**Published:** 2015-11-25

**Authors:** M. Benavides, A. Antón, J. Gallego, M. A. Gómez, A. Jiménez-Gordo, A. La Casta, B. Laquente, T. Macarulla, J. R. Rodríguez-Mowbray, J. Maurel

**Affiliations:** Hospital Universitario Regional y Virgen de la Victoria, Málaga, Spain; Hospital Universitario Miguel Servet, Zaragoza, Spain; Hospital General Universitario, Elche, Spain; Hospital Universitario Reina Sofía, Córdoba, Spain; Hospital Universitario Infanta Sofía, San Sebastián de los Reyes, Madrid, Spain; Hospital Universitario Donostia, San Sebastián, Spain; Institut Català d’Oncologia L’Hospitalet, Barcelona, Spain; Hospital Universitario Vall d’Hebron e Instituto de Oncología Vall d’Hebron, Barcelona, Spain; Hospital Infanta Cristina, Badajoz, Spain; Hospital Clínic, Barcelona, Spain

**Keywords:** Biliary tract cancer, Gall bladder cancer, Cholangiocarcinoma

## Abstract

Biliary tract cancer (BTC) is an uncommon and highly fatal malignancy. It is composed of three main different entities; Gall bladder carcinoma (GBC), intrahepatic cholangiocarcinoma (iCC) and extrahepatic cholangiocarcinoma (eCC) sharing different genetic, risk factors and clinical presentation. Multidetector-row computed tomography (MDCT) and magnetic resonance cholangio-pancreatography (MRCP) are the more important diagnostic techniques. Surgery is the only potentially curative therapy but disease recurrence is frequent. Treatment with chemotherapy, radiotherapy or both has not demonstrated survival benefit in the adjuvant setting. Cisplatin plus gemcitabine constitutes the gold standard in metastatic disease. New ongoing studies mainly in the adjuvant and neoadjuvant setting along with molecular research will hopefully help to improve survival and quality of life of this disease.

## Introduction

Gallbladder cancer (GBC) and cholangiocarcinoma (CC) are distinct entities with different epidemiology, biology and clinical presentation. CC is classified as intrahepatic (iCC) and extrahepatic (eCC). These recommendations include epidemiology, diagnostic and staging procedures, biology and therapeutic aspects. Studies used as a basis for these guidelines are graded according to the Oxford Center for Evidence-based Medicine levels.

## Epidemiology

Cholangiocarcinoma (CC) is the second most common primary liver cancer after hepatocellular carcinoma and is best classified anatomically as intrahepatic (iCC), and extrahepatic (eCC). eCC occurs anywhere within the extrahepatic bile duct, including the intrapancreatic portion and are further classified into hilar/perihilar (pCC, also called Klastkin tumors), or distal (dCC). pCC is the most common type of CC, followed by dCC and then the intrahepatic forms. The incidence of iCC has increased over the past three decades while the incidence of perihilar and distal extrahepatic cholangiocarcinoma has remained stable [1, 2]. The prognosis is dismal owing to its silent clinical character, difficulties in early diagnosis and limited therapeutic approaches.

GBC is the most common and aggressive type of all the BTCs and the vast majority are adenocarcinoma with incidence steadily increasing with age. It is characterized by local and vascular invasion, extensive regional lymph node metastases and distant metastases [3].

## Risk factors

An overview of risk factors for CC and GBC is presented in Table 1 [2, 4]. Hepatolithiasis, primary sclerosing cholangitis, liver flukes, biliary duct cysts, specific toxins and inflammatory bowel disease are the major risk factors for CC. A systematic review and meta-analysis reveal that hepatitis C virus is associated with a significantly increased risk of iCC and eCC.

**Table 1:** Risk factors [2, 4]

Cholangiocarcinoma	LE Ia	LE IIb
General risk factors	Age >65 Excessive alcohol intake	Tobacco smoking Obesity Type II diabetes
Congenital risk factors	Caroli’s disease choledochal cysts, congenital hepatic fibrosis	
Viral risk factors	VHB, VHC,	
Inflammatory risk factors	Primary schlerosis cholangitis, hepatolithiasis, cirrhosis, inflammatory bowel disease, biliary enteric-anastomosis	
Parasitic risk factors		Clonorchis sinensis Opisthorchis viverrini
Chemical risk factors	Thorotrast	Nitrosamines, vinyl chloride, dioxin, oral contraceptives, isoniazid, asbestos
Gallbladder cancer		
Inflammatory risk factors	Chronic Cholecystitis and Gallstones	Porcelain gallbladder Inflammatory bowel disease
Congenital risk factors		Anomalous junction of the pancreato-biliary duct
Bacterial risk factors	*Escherichia coli*, chronic carriers of *Salmonella typhi* or *paratyphi*	
Others risk factors	Gallbladder polyps, obesity	

*LE* level of evidence, *VHB* hepatitis B virus, *VHC* hepatitis C virus

## Staging

The clinical presentation of GBC often mimics biliary colic or chronic colecystitis. Hence, it is not uncommon to be an incidental finding at cholecystectomy for a benign gallbladder disease. Other possible clinical presentations are: suspicious mass on ultrasound or biliary tract obstruction with jaundice. Tumor markers, in particular serum CA 19–9 determination, can be helpful but are not diagnostic. Liver function tests and assessment of hepatic reserve are mandatory in patients candidates for surgical resection.

### Imaging studies

Technical advances, such as MDCT have significantly improved the accuracy in diagnoses. Thoracic and abdominal-pelvic MDCT is the standard technique to rule out metastatic disease. MDCT and MRCP are both adequate to evaluate vascular invasion (portal and hepatic artery involvement/encasement) [5, 6] (Level of Evidence IIa, Grade of Recommendation A). PET-CT may be considered to rule out metastatic disease in patients without metastatic spread on MDCT, but remains investigational [7] (Level of Evidence IIIb, Grade of Recommendation C).

### Pathological diagnosis

A preoperative biopsy is not always needed before proceeding with a definitive curative resection. Pathological diagnosis is mandatory for all patients undergoing systemic chemotherapy. Core biopsies are required for definitive diagnosis. The expression of cytokeratin 7 and 19 and the absence of cytokeratin 20 may be helpful to establish a biliary origin.

### Unresectability criteria

Contraindications for iCC surgery is multifocal presentation and for iCC, eCC and GBC surgery are vascular invasion of main hepatic artery, portal vein encasement or invasion of both branches of hepatic artery or portal vein, distant lymph nodes (celiac trunk, mesenteric artery and peri-aortic nodes) and obviously distant metastasis.

## Biology: genetic and molecular features

A large number of genetic alterations have been described in BTCs. The induction of different genetic profiles could be driven by different carcinogenic factors, location or histological subtypes. Therefore, heterogeneous sample sets and different technologies used to detect mutations can explain some difference in the results obtained. Recently, high-throughput next-generation sequencing has enabled mutational profiling of BTC, providing new insights into the genetic basis of tumorigenesis [8–10].

Table 2 shows more frequent genetic alterations described in BTCs [3, 11–13]. Some of them have high variability among authors. Additional genes have been implicated in BTC carcinogenesis, including STK11 (LKB1), PBRM1 9 % IDH-1/2, 22 %). Loss of heterocygosity, hypermethylation and other features related with inflammation as overexpression of COX-2 have been described in the literature [14].

**Table 2:** Genetic alterations frequently described in BTCs [3, 11–13]

	More frequent GA	BTCs (%)	iCC (%)	eCC (%)	GBC (%)
GA/patient	Mutations		2.9	4.4	4
K-Ras	Codon 12 mutation	17–54	4–54	10–42	11–25
B-RAF	Mutation (exclusive of K-Ras mutation)		5–22	3	1–33
EGFR	Mutation Amplification		10–20 27	5–18 19	9–12
Her-2	Amplification		0–3	5–25	11–16
P53	Mutation (exon 5) Amplification	35–44	37	33–45	36
PTEN	Amplification	15			
PI3K/TOR	Mutation/others^a^		5–9	0–14	4–15
SMAD4	Mutation	16	3.6–13	16–55	
IDH1/2	Mutations	18/5	18–23.6	0	0
BAP1		25	9	10	
ARID1A		12–20	17–20	5–12	13
CDKN2 A/B		5.6–15 17–19	18–88	17–55	19–62
FGFR	Mutations/translocations		11–12.7	0–5	3
MET	Activation		4	0	0

*GA* genetic alterations

^a^Pathway implied in 25 % of iCC and 40 % eCC but only 6 % mutations described

### Molecular classification of BTCs

Andersen et al. [15] performed a genomic analysis of 104 resected cholangiocarcinomas. They identify four survival subtypes (SGI-IV). SGIII was characterized by gene associated with proteosomal activity and the worst prognosis.

Sia et al. [16] performed an integrative genomic analysis of 149 iCC samples. They identify two subgroups of iCC:

- Inflammmation class (38 %): Characterized by activation of inflammatory signalling pathways (overexpression of cytokines, and STAT3 activation).
- Proliferation class (62 %): Characterized by the activation of oncogenic signalling pathways (RAS, MAPK, KRAS, BRAF, and MET). This group was enriched with a gene expression signature associated with reduced survival.

In conclusion, different histological subtypes and tumour locations are associated with specific genetic alterations (Level of Evidence IIb). The most frequent alterations are prognostic markers but not targetable (*KRAS, p53*), but some infrequent genetic alterations are targetable (*BRAF, FGFR2, IDH1*). Different classes of iCC based on molecular features might require different treatment approaches.

Up to date, several specific molecular treatments are under evaluation in clinical trials that target BTCs with specific characteristics, as FIG-ROS fusions, EGFR mutations, IDH1 mutations, FGFR2, BRAF mutations and others. Better knowledge of specific genetic or molecular abnormalities offers potential for individualized treatment of patients with BTCs in the near future (Level of Evidence IIb, Grade of Recommendation C).

## Treatment

### Localized disease

#### Surgery

##### Gallbladder cancer

GBC is suspected preoperatively in only 30–40 % and in 60–70 % is discovered incidentally after cholecystectomy for other reasons, on pathologic review. Patients with T1b, T2, T3 disease that are incidentally identified in a cholecystectomy specimen should undergo re-resection which includes adequate lymphadenectomy including regional nodes and a goal recovery of at least 6 nodes.

##### Intra and extrahepatic cholangiocarcinoma

Surgical resection is the only strategy with the potential for cure. The extent of liver resection depends of the function of remnant liver. Partial hepatectomy remains the mainstay of curative treatment for iCC and patients with potentially resectable tumors with ≤3 cm of diameter but without adequate liver function for hepatic resection, can be considered for ablation. The role of routine lymphadenectomy is not defined. For eCC, resection should include extrahepatic bile duct, regional lymphadenectomy and hepatectomy of the right or left lobe (Level of Evidence IIa, Grade of Recommendation A).

#### Prognostic factors in resectable BTCs

In iCC, age, lymph node metastases, vascular invasion, tumor size and multiple tumors are clinically relevant variables [17, 18]. Five-year OS ranges between 30 and 40 %. In eCC, lymph nodes, microscopically residual tumor and tumor grade differentiation are relevant variables. Five-year OS ranges between 20 and 30 % [19]. Finally, important prognostic factors for resected GBC are T and N staging. Five-year OS ranges between 10 and 30 % [20].

Prognostic factors are useful to optimize clinical trials design in the adjuvant setting. Because prognostic factors and survival are different in different tumors types (GBC, iCC and eCC), adequately powered randomized studies should be optimally conducted separately in the three different locations (Level of Evidence Ia, Grade of Recommendation A).

#### Adjuvant therapy

A systematic review and meta-analysis of 20 studies including 6712 resected BTCs patients (GBC, iCC, eCC), assessed the impact of chemotherapy, radiation or both in the adjuvant setting. There was a non-significant improvement in overall survival with adjuvant treatment compared with surgery alone and these results were also non-significant when analyzed independently for patients with GBC, iCC or eCC [21]. It should be noted that only one phase III trial was included in the meta-analysis. Nonetheless and because of the grim prognosis, adjuvant therapy is often recommended in clinical practice in patients with high-risk features (node and/or margin-positive). Ongoing randomized studies will define the role of adjuvant therapy in BTCs but current data do not support its use after BTC resection (Level of Evidence IIb, Grade of Recommendation C).

#### Follow-up

For BTCs there is currently no evidence that regular follow-up influences outcome. In case a routine follow-up was recommended, this should be restricted to history and physical examination. Image and laboratory tests should only be performed under suspicion (Level of Evidence IV, Grade of Recommendation D).

### Locally advanced disease

BTCs are often diagnosed at an advanced stage defined as unresectable disease (metastatic or locally advanced) due to their non-specific symptomatology. Locoregional therapies for unresectable iCC have been evaluated in small studies including strategies such as radiofrequency ablation, transarterial chemoembolization, drug-eluting bead, and transarterial radioembolization. There are also studies evaluating the role of chemoradiation in advanced BTC. Nevertheless the magnitude of benefit of all these options vs systemic chemotherapy is currently unknown (Level of Evidence IIb, Grade of Recommendation C).

### Metastatic disease

Given its rarity and diversity, few clinical trials have studied optimum treatment for BTC. Treatments for these cancers have been extrapolated from regimens for metastatic pancreatic cancer. However, as of 2010, many new trials have been designed to achieve optimum chemotherapeutic treatment for advanced BTC. In 2007 Eckel et al. [22] published a pooled analysis of 104 trials in order to pioneer a standard of care. With more than 2800 patients, this pooled analysis suggested that the combination of gemcitabine and cisplatin or oxaliplatin was the most active regimen. These results were later confirmed in the Indian monocentric randomized trial exclusively including gallbladder cancer [23].

The British United Kingdom ABC-02 is so far the largest published trial designed for locally advanced or metastatic BTC [24]. This study published in 2010 randomized 410 ECOG 0–2 patients to treatment with gemcitabine (G) versus gemcitabine in combination with cisplatin (GC), being overall survival the primary endpoint. After a median follow-up of 8.2 months, the median overall survival was 11.7 months in the GC group and 8.1 months in the G group (HR 0.64; CI 99 %, 0.52–0.80; *p* < 0.001). The median progression-free survival was 8.0 months in the GC group and 5 months in the G group (*p* < 0.001). In addition, the rate of tumor control among patients in the GC group was significantly increased (81.4 vs 71.8 %, *p* = 0.049). Adverse events were similar in the two groups, with the exception of more neutropenia in the GC group. Another Japanese study [25] and the meta-analysis carried out with them [26] recommended the combination of GC as standard of care for the first-line treatment of advanced BTCs in patients with good performance status (Level of Evidence I, Grade of Recommendation A).

In patients with poor performance status (ECOG > 2) only best supportive care is indicated. A systematic literature review including phase II trials, retrospective analyses and case reports [27] showed that there is insufficient evidence to recommend second-line chemotherapy in advanced BTC. Factors such as performance status, CA 19.9 value, progression-free survival after first-line and previous surgery of primary tumor may allow adequately individual patient-risk stratification in prospective randomized trials [28] (Table 3) (Level of Evidence IIb, Grade of Recommendation C).

**Table 3:** Multivariate prognostic model for second-line chemotherapy in advanced BTC [28]

Factors	PFS after first line CT ≥6 months
	Previous surgery on primary tumor
	Pretreatment CA19.9 ≤152 U
	ECOG performance status 0
Prognostic Groups	
Good-risk	0–1, negative prognostic factors
Intermediate-risk	2, negative prognostic factors
Poor-risk	3–4, negative prognostic factors
Median OS (months)	
Good-risk	13.1 (CI 95 % 9–17.2)
Intermediate-risk	6.6 (CI 95 % 5.2–8.0)
Poor-risk	3.7 (CI 95 % 3.0–4.4)

*PFS* progression-free survival, *CT* chemotherapy, *OS* overall survival

## Conclusions

**Fig. 1:**
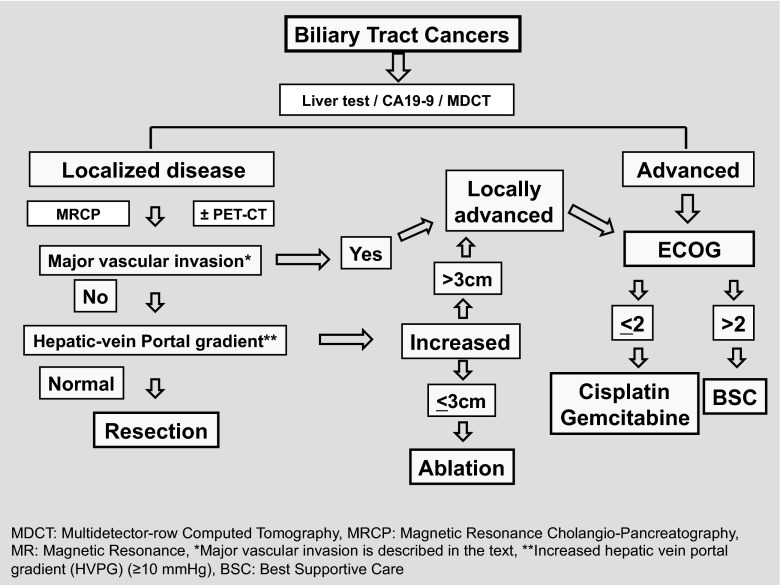
Diagnostic work-up and treatment guidelines

BTCs are uncommon and poorly understood tumours with a prominent geographic variability. Recognized risk factors are being thought to be contributory although high scientific evidence is generally missing. Different clinical presentations are seen according to the primary tumour location with chronic inflammation and cholestasis of the bile ducts frequently associated. Figure 1 summarizes diagnostic work-up and treatment guidelines.

Complete surgical resection is the only potentially curative therapy but disease recurrence is frequent. The benefit of adjuvant therapy following surgery (R0 or R1) for BTCs (GBC, iCC and eCC) is currently unknown and highlights the need for well-designed randomized trials to answer this question. Follow-up is not evidence based because no data support the benefit.

In locally advanced disease, the goal of palliation is relief of symptoms (pain/jaundice) along with prolongation of life. Treatment with chemotherapy, radiotherapy or local therapies (chemo and radioembolization) has been studied but results from randomized trials are currently not available. In metastatic disease, cisplatin plus gemcitabine is the gold standard based in two phase III trials and there is no scientific support for second-line therapy. Currently ongoing clinical trials of targeted therapies requiring specific mutations (BRAF, ALK, FGFR2, EGFR, VEGFR, IDH1-2, ROS1) may clarify the value of different targeted therapies.

New ongoing studies mainly in the adjuvant setting along with new and important research related with the underlying molecular aberrations of BTCs, will hopefully help to improve survival and quality of life of these diseases.
